# Research priorities for Long Covid: refined through an international multi-stakeholder forum

**DOI:** 10.1186/s12916-021-01947-0

**Published:** 2021-03-31

**Authors:** Gail Carson, Long Covid Forum Group, Gail Carson, Louise Sigfrid, Piero Olliaro, Alice Norton, Giuseppe Paparella, Romans Matulevics, Annelies Gillesen, Peter Horby, Claire Hastie, Margaret O’Hara, Jake Suett, Sarah Moore, Richard Vaux, Jean Marie Habarugira, Genevieve Boily-Larouche, Ella Clark, Peter Hart, Josie Golding, Claire Madelaine, Brian Mackenwells, Kristina Ruze, Nina Jamieson, Liliana Resende, Evelyn Depoortere, Anna Kinsey, Daniel Munblit, Fahmy Hanna, Janet Diaz, Annelies Wilder-Smith, Charles Wiysonge, Charu Kaushic, Luis Felipe Reyes, Roberto Bruzzone, Yazdan Yazdanpanah, Tedros A. Ghebreyesus, Alex van Blydenstein, Alpha Keita, Andre Siqueira, Angela Cheung, Charitini Stavropoulou, Frances Simpson, Janet Scott, Joseph Breen, Joseph Fokam, Ken Baillie, M. Netravathi, Margaret Herridge, Nisreen Alwan, Priscilla Rupali, Ryan Zarychanski, Seong-Ho Choi, Lwazi Mlaba, Moses Badio, Bhasha Mewar, Gina Assaf, Christopher Brightling, Danny Altmann, Fernando Bozza, Ivan Hung, Norio Ohmagari, Sally Singh, Shinichiro Morioka, Simon Hatcher, Simone Piva

**Affiliations:** University of Oxford, ISARIC and GloPID R Secretariat, Oxford, UK

**Keywords:** Long Covid, Coronavirus research, WHO R&D COVID-19 Research Agenda, GloPID-R, ISARIC, Long Covid Support Group

## Background

Coronavirus disease 2019 (COVID-19) can lead to a diverse range of clinical manifestations, ranging from an asymptomatic infection to an acute respiratory distress syndrome, and multiorgan failure with high mortality rates [1]. It is established that SARS-CoV-2 not only infects the respiratory tract but that the ensuing viral replication and immune response also affects multiple organ systems, in addition to an acute systemic inflammatory response and in some cases accompanying tissue hypoxia and shock.

While many who have been infected have uncomplicated recoveries, some have prolonged illness. Prolonged course of illness has been reported in adults and children and is affecting both those who were hospitalised with COVID-19 and those who were not [2–7].

In December 2020 ISARIC (the International Severe Acute Respiratory and emerging Infection Consortium), the research funders group GloPID-R (The Global Research Collaboration for Infectious Disease Preparedness) and global group, Long Covid Support, jointly organised a ‘Long Covid Forum’ [8]. This public forum aimed to gain a better understanding of ‘Long Covid’ and to define research priorities for funders and researchers to take forward.

## Emerging themes

### Global patient voices

A complex, multi-faceted condition involving a range of physical, cognitive, psychological and social implications was described by people living with Long Covid from around the world. There was a call for a systematic way to define cases and to build awareness of Long Covid in the medical community globally to ensure recognition. Themes of isolation, social stigma, inability to care for family and economic repercussions were heard repeatedly. Integration of multi-disciplinary, holistic care into health systems is needed, including understanding the scale of resources that need to be mobilised and how to ensure equity in access to care across the world. Specific questions emerged around whether anti-viral treatment, during acute infection, could prevent Long Covid and improve long-term outcomes. Pertinent questions about the need to understand the effect that vaccination may have on people living with Long Covid were raised.

### Overarching summary of knowledge of Long Covid

An analysis by a rapid living systematic review on Long Covid clinical characteristics [7] highlights the limited evidence base and heterogeneity in the design of published studies. Importantly, this work indicates little difference in the severity of symptoms between the studies in hospitalised and non-hospitalised groups. An analysis from the UKCDR and GloPID-R COVID Research Project Tracker [9] similarly highlighted little ongoing research on Long Covid with few studies in non-hospitalised groups or multi-country studies and no studies in children. A clear need for further studies in mild infection cases, including those who did not receive PCR confirmation, children and young people, and studies in different resourced settings using standardised protocols, risk factors and endpoints was identified to inform clinical and public health management, rehabilitation and support.

### Country experiences

Research presented from around the world highlighted heterogeneity in Long Covid presentation, which may be linked to different treatment responses in the acute phase of infection. Risk factors—genetic, environmental, co-morbidities or social—for Long Covid need defining as well as the impacts of chromic co-infections (e.g. with HIV and TB) and co-morbidities on disease outcomes.

### Reflecting on Chikungunya

There are important similarities between Chikungunya and COVID-19, including long-lasting and heterogenous symptoms post-acute infection and research challenges, due to a lack of standardisation of case definitions, study measures and inclusion criteria. The importance of investigating both social and health implications of prolonged symptoms was highlighted. There was a recognition of being prepared to research into the longer-term effects of any emerging pathogen.

### Ongoing Long Covid studies

Emerging results from ongoing research from a range of countries were presented and discussed to identify multiple remaining research gaps. These included the need to understand the mechanism of pathogenesis; the immune response and immune system dynamics (given the divergent working hypotheses of a failure of adaptive immunity to respond enough vs autoimmunity, where a prolonged or exaggerated response could be damaging); inclusion of wider populations, including people who were never hospitalised or even tested; animal and tissue models; and genetic host factor characterisation. There is a gap in case-control and interventional studies and an urgency to understand the aetiology, identify treatments and develop holistic care pathway for rehabilitation, interventions and social support systems.

### Psychosocial health

Few studies have focussed on the psychosocial impact of COVID-19 to date and most lack a comparative group. Studies indicate that up to a fourth of patients experience neurological and psychosocial sequelae including depression and anxiety; the mechanisms underlying SARS-CoV-2 infection and its effects on the nervous system need to be explored to identify the interplay between neurological symptoms, including those manifesting as psychological symptoms, the virus and the immune response. Case-control studies, with matched control groups within the current pandemic context, are urgently needed.

## Discussion and recommendations

Further action is clearly needed to make sure that Long Covid does not become the long-lasting legacy of the pandemic with potentially millions of people suffering, with an impact on health and social care systems and wide, long-lasting socioeconomic consequences. Although there is emerging recognition of Long Covid, the evidence base is limited and fragmented and many research questions remain. The key pressing research questions are summarised in Table 1 aligned against WHO priorities [10].

**Table 1:** Research priorities identified at the Long Covid Forum. Framework for identifying research needs from the Long COVID Forum

Research priorities (aligned to the WHO mid-term and long-term research priorities: 2019 novel coronavirus [4])	Identified sub-priority for Long Covid	Populations (multi-country studies needed)
**Virus: natural history, transmission and diagnosis**	- Identify pathogenesis - Investigate the impact of chronic and acute co-infections	Hospitalised patients; non-hospitalised patients; clinical diagnosis only, individuals; children; vulnerable communities; resource-constrained populations
**Epidemiological studies**	- Define the clinical presentations of Long Covid and characterise the burden and spectrum of Long Covid across populations according to clear case definition - Determine any associations between host genetic factors and Long Covid	
**Clinical characterisation and management**	- Agree case definition and diagnosis - Establish causality -Relationship between acute disease and Long Covid development - Describe underlying mechanisms to identify potential therapeutic targets - Investigate pathogenesis to explain, e.g. thrombotic tendencies, organ impairment - Investigate the impact of chronic and acute co-infections and co-morbidities - Characterise mental health and neurological impacts	
**Candidate therapeutics R&D**	- Investigate antiviral and anti-inflammatory therapeutics and therapeutic timings to prevent Long Covid - Investigate therapeutics to treat Long Covid symptoms and non-pharmaceutical interventions	
**Candidate vaccines R&D**	- Investigate the impact of COVID-19 vaccination on people with Long Covid - Investigate whether vaccination prevents Long Covid - Investigate re-infection in people with Long Covid and impact on vaccine priority list	
**Social sciences in the outbreak response**	- Health systems research on identifying and supporting Long Covid cases (through holistic care)	

Reorienting and including Long Covid in existing cohorts, trials and studies might be the most efficient way to undertake this research globally and strengthen health systems for future emerging pathogens.

## Conclusion

Research on Long Covid will require a multi-disciplinary and globally coordinated approach that supports harmonised and large-scale case-control and interventional studies that have the power to provide quality evidence to inform policy and patient care across the full range of populations and countries affected.

Governments of the world need to ensure the strengthening of health systems to be able to provide treatment, support and rehabilitation to improve long-term COVID-19 outcomes. To prevent Long Covid becoming the pandemic of 2021, controlling the high rates of infection has to remain a global priority.

## Data Availability

Not applicable
